# Supplementary material for: The percentile-matching technique for synthetic eye tracking signal degradation: A biometric case study
Source: PLoS One. 2025 Nov 19;20(11):e0334605. doi: 10.1371/journal.pone.0334605 (PMC12629438; doi:10.1371/journal.pone.0334605)
Supplement: Attachment — Submitted filename: Rebuttal_Letter.docx [file pone.0334605.s001.docx]

**PONE-D-24-31041**

**The percentile-matching technique for synthetic eye tracking signal degradation: a biometric case study**

Dear Editor,

Please find the response to each of the comments below:

1. Please ensure that your manuscript meets PLOS ONE's style requirements, including those for file naming. The PLOS ONE style templates can be found at 
<https://journals.plos.org/plosone/s/file?id=wjVg/PLOSOne_formatting_sample_main_body.pdf> and 
<https://journals.plos.org/plosone/s/file?id=ba62/PLOSOne_formatting_sample_title_authors_affiliations.pdf>

Our manuscript follows PLOS ONE’s style requirements.

2. Thank you for stating the following financial disclosure: 
"Authors Samantha Aziz and Dillon Lohr are recipients of National Science Foundation Graduate Research Fellowship under Grant No. DGE-1840989 and DGE-1144466, respectively."

Please state what role the funders took in the study.  If the funders had no role, please state: "The funders had no role in study design, data collection and analysis, decision to publish, or preparation of the manuscript." 
If this statement is not correct you must amend it as needed. 
Please include this amended Role of Funder statement in your cover letter; we will change the online submission form on your behalf.

Addressed in the cover letter.

3. Thank you for stating the following in the Acknowledgments Section of your manuscript: 
"Authors Samantha Aziz and Dillon Lohr are recipients of National Science Foundation 
Graduate Research Fellowship under Grant No. DGE-1840989 and DGE-1144466, 
respectively. Any opinion, findings, and conclusions or recommendations expressed in 
this material are those of the authors(s) and do not necessarily reflect the views of the
National Science Foundation. "

We note that you have provided funding information that is  currently declared in your Funding Statement. However, funding information should not appear in the Acknowledgments section or other areas of your manuscript. We will only publish funding information present in the Funding Statement section of the online submission form. 
Please remove any funding-related text from the manuscript and let us know how you would like to update your Funding Statement. Currently, your Funding Statement reads as follows: 
"Authors Samantha Aziz and Dillon Lohr are recipients of National Science Foundation Graduate Research Fellowship under Grant No. DGE-1840989 and DGE-1144466, respectively."

Please include your amended statements within your cover letter; we will change the online submission form on your behalf.

We have removed the funding information from the manuscript and amended statements have been added in the cover letter.

4. Please review your reference list to ensure that it is complete and correct. If you have cited papers that have been retracted, please include the rationale for doing so in the manuscript text, or remove these references and replace them with relevant current references. Any changes to the reference list should be mentioned in the rebuttal letter that accompanies your revised manuscript. If you need to cite a retracted article, indicate the article’s retracted status in the References list and also include a citation and full reference for the retraction notice.

None of our cited papers have been retracted. We reviewed the reference list.

Additional Editor Comments:

This study demonstrates an improved model-based approach for synthetic degradation of pre-recorded eye movement signals. I reckon that the issue of eye tracking and like any other biometric are still contentious about there applications especially in end-user authentication due to varying laws and the tendencies of being lost and used for malicious activities.

Thank you.

The addition to knowledge of this study range from the improvement on data degradation using synthetic data for a case study. All the same, there was no information on subjecting this outcome to long-term/simulated integrity test. This work to the best of my knowledge followed known scientific rigours in carrying out the experiment and result presentation and reporting.

Thank you.

What I found largely missing or confused is why the authors used a modified model as benchmark, when it has not been tested and proven to be trusted.

The mechanistic data degradation model described in the paper titled – “Using machine learning to detect events in eye-tracking data” was implemented herein for initial benchmarking purposes. While it was attempted to replicate the implementation as closely as possible to the procedure described in the original manuscript, some modifications were made to enhance performance for the current application as described in the section- Benchmark transformation model in the manuscript.

Zemblys R, Niehorster DC, Komogortsev O, Holmqvist K. Using machine learning to detect events in eye-tracking data. Behavior research methods. 2018;50(1):160–181.

Also, there is the need to comparatively compare the results with existing literature to provide through empirical evidence the addition to knowledge and contributions to the scientific community. Doing this help to situate the work properly for further studies by an interested party. Nevertheless, I commend the authors on their efforts.

In table 1 of the manuscript, we compare the results in terms of three metrics. Also In figure 4 we compare the results between baseline and modified versions from the biometric perspective.
