# Supplementary material for: The percentile-matching technique for synthetic eye tracking signal degradation: A biometric case study
Source: PLoS One. 2025 Nov 19;20(11):e0334605. doi: 10.1371/journal.pone.0334605 (PMC12629438; doi:10.1371/journal.pone.0334605)
Supplement: Attachment — Submitted filename: Rebuttal_Letter_V2.docx [file pone.0334605.s002.docx]

**PONE-D-24-31041**

**The percentile-matching technique for synthetic eye tracking signal degradation: a biometric case study**

Dear Editor,

Please find the response to each of the comments below:

Reviewer #2: 1. The clarity of the figures in the manuscript is insufficient, so vector figures are recommended.

Thank you for pointing this out. We have replaced figures with high-resolution vector (in PDF/SVG format) to enhance clarity and ensure better readability.

2. The structure of the manuscript is somewhat confusing. Should the data set be described in the experimental section?

Thank you for your comments. We would like to clarify that our manuscript does not contain a section explicitly titled "Experimental Section." Currently, the dataset details are provided within the Methods section, which we intended to include both methodological and experimental setup information. If the reviewer recommends separating this into a distinct "Experimental" section for clarity, we are happy to make that structural adjustment. We would appreciate further guidance on how the reviewer would prefer this information to be organized.

3. Some related works on target tracking tasks should be discussed in this paper: --Self-supervised deep correlation tracking, --Aligned Spatial-Temporal Memory Network for Thermal Infrared Target Tracking, --Active learning for deep tracking…

Thank you for these suggestions. While these works represent important advancements in generic object tracking under RGB or thermal modalities, they differ considerably from the focus of our work.

4. Experiment validations are not convincing. Some additional experiments need to be conducted to make its conclusion stronger.

We appreciate the reviewer’s concern regarding the strength of the experimental validation. To better address this point and ensure our revisions align with the reviewer’s expectations, we would greatly value any specific suggestions or directions regarding the types of additional experiments that would most effectively strengthen our conclusions.
